# Evaluation of podocin in urine in horses using qualitative and quantitative methods

**DOI:** 10.1371/journal.pone.0240586

**Published:** 2020-10-15

**Authors:** Natalia Siwińska, Urszula Pasławska, Remigiusz Bąchor, Barbara Szczepankiewicz, Agnieszka Żak, Paulina Grocholska, Zbigniew Szewczuk

**Affiliations:** 1 Department of Internal Diseases with Clinic for Horses, Dogs and Cats, Faculty of Veterinary Medicine, Wrocław University of Environmental and Life Sciences, Wrocław, Poland; 2 Veterinary Institute, Faculty of Biological and Veterinary Sciences, Nicolaus Copernicus University, Toruń, Poland; 3 Faculty of Chemistry, University of Wrocław, Wrocław, Poland; Institut National de la Santeet de la Recherche Medicale (INSERM), FRANCE

## Abstract

No sensitive method for diagnosing early kidney dysfunction in horses has been identified so far. Many studies carried out in humans and small animals show that podocin can be useful to diagnose various kidney diseases, mainly affecting the glomeruli. The aim of this study was to perform a qualitative and quantitative analysis of podocin in urine samples obtained from healthy horses, horses with clinical kidney dysfunction and horses at risk of acute kidney injury. The study objectives aimed to assess: (1) whether the selected podocin tryptic peptide for LC-MS-MRM allows for podocin detection in horse; and (2) whether the species-specific ELISA test makes this detection possible as well;, (3) whether the chosen methods are sensitive enough to detect kidney dysfunction and glomerular injury, (4) whether the results of the tests applying both methods correspond with one another, (5) whether the results correlate with the hematological and biochemical data. The signals that may indicate the presence of trypsin fragments of podocin were found in three healthy horses, all the horses diagnosed with kidney dysfunction and half of the animals at risk for acute kidney injury. The concentration of podocin, diagnosed with the ELISA test was as follows: from 0.19 to 1.2 ng/ml in healthy animals, from 0.19 to 20.0 ng/ml in AKI horses, from 0.29 to 5.71 ng/ml in horses at risk for acute kidney injury. The results of both methods corresponded significantly. Podocin may be a potential biomarker of clinical kidney disease in horses and may be used in the detection of glomerular injury. However, its use is limited by the possibility of physiological podocyturia. LC-MS-MRM seems to be a more sensitive method to evaluate the presence of podocin than the ELISA test, whilst selected tryptic peptides of podocin appear to apply to horses. The ELISA test showed greater effectiveness in excluding the disease than in confirming it.

## Introduction

The current diagnosis of kidney disease in horses is mainly based on the presence of elevated serum creatinine. This increase is observed when more than 50% of the organ is dysfunctional [1]. There is still a lack of sensitive methods to detect early kidney disease, like acute kidney injury (AKI) in horses. Numerous studies have shown that podocyturia can be an excellent diagnostic indicator of many kidney diseases in human and small animal medicine [2–10]. Podocytes (or visceral epithelial cells) are highly specialized glomerular epithelial cells, lining the outer surface of the glomerular capillaries. They play an active role in preventing plasma proteins from entering the urinary ultrafiltrate by providing a barrier comprising filtration slits [11]. They are exclusively found in glomeruli and have a minimal proliferative capacity *in vivo* [12, 13]. Due to their location, they are vulnerable to damage from toxins, immune complexes, inflammatory processes or mechanical forces such as the filtration pressure [14, 15]. In addition, many drugs can damage podocytes, causing podocyturia and increased permeability of the glomerulus to large molecules [16, 17]. The presence of podocytes in urine is determined by the detection of podocyte-associated molecules, such as podocin, nephrin or synaptopodin, of which podocin seems to have the most possibilities, according to literature [2, 4, 16, 18]. Podocin is a protein present exclusively in podocytes, especially near the slit diaphragms, and may be released in urine following podocyte damage [16, 19, 20]. According to literature, podocin can be detected in urine using qualitative methods such as liquid chromatography-mass spectrometry in multiple reaction monitoring mode (LC-MS-MRM) or quantitatively using immunoassays such as the enzyme-linked immunosorbent assay (ELISA) [16, 21–24]. However, to date, no method of podocin detection in equine urine samples has been described. ELISA is a simple, well-known and widely available method that allows for detecting and quantitating antibodies or antigens. It is used to detect many proteins (including podocin) in human and animal medicine [23, 24]. Another method, LC-MS-MRM, which was applied in this study, has been well described and recommended in humans and small animals to detect selected podocin tryptic peptides [6, 16, 21, 22]. Very recently, the authors have tested the applicability of the LC-MS-MRM method to identify the podocin tryptic peptide in feline [21] and canine [22] urine samples collected from animals with diagnosed chronic kidney disease and with glomerular injury (cardiorenal syndrome due to myxomatous mitral valve disease). The obtained results confirmed the applicability of the LC-MS-MRM method in the analysis of podocin in urine samples. Based on the previous results on small animals and considering that there are no species-specific biomarkers to diagnose or confirm early kidney disease, further research was conducted using equine urine samples. Horses are predisposed to developing acute kidney dysfunction due to the relatively frequent use of nephrotoxic drugs in daily veterinary practice (like nonsteroidal anti-inflammatory drugs–NSAID or aminoglycoside antibiotics–gentamicin) and exposure to the development of hypovolemia and endotoxemia (gastrointestinal diseases) [25–31]. According to the above literature data, horses more frequently develop acute tubular damage after exposure to the above mentioned factors, whilst glomerular injury is considered rare. Noninvasive diagnosis of glomerular injury is based on the detection of proteinuria or increased urine protein to creatinine ratio [32]. Exercise can cause transient proteinuria and the presence of blood in the urine can falsify the urine protein to creatinine ratio, which makes diagnosis difficult, especially in non-azotemic cases [33, 34]. The final diagnosis is made on the basis of a histopathological examination. However, kidney biopsy is invasive and may lead to serious complications [35]. Therefore, the glomerular injury in early kidney diseases in these animals may be difficult to estimate due to the lack of sensitive biomarker.

The aim of this study was to perform a qualitative and quantitative analysis of podocin in urine samples obtained from healthy horses, horses with clinical kidney dysfunction and non-azotemic horses at risk for AKI. The study objectives included: (1) determining whether the selected sequence of the tryptic podocin peptide for LC-MS-MRM can be used for detecting podocin in horses; and (2) whether the species-specific ELISA test is useful for this purpose, (3) whether the chosen methods are sensitive enough to detect kidney dysfunction and glomerular injury, (4) whether the results of both methods correspond with one another, (5) whether the results correlate with the hematological and biochemical data.

## Materials and methods

### Animals

The study was conducted on 71 adult warmblood horses of different breeds. The animals were divided into three groups. The first group served as the negative control group and consisted of healthy horses, the second formed the positive control group and included horses with a clinical form of AKI. The third group contained horses at risk for AKI but without signs of kidney disease.

Control group I consisted of 30 healthy horses (15 females and 15 males) between 3 and 29 years old (mean 14.7 SD 8.4), weight 554 SD 80 kg, which were preventively examined with the consent of the owners. Inclusion criteria in this group were: no past or present urinary or cardiovascular disease; no current clinical signs of local or general diseases and no history of such illness for at least 6 months before and 6 months after the study; no drug administration in the 6 months prior to the start of the study; normal results of blood (complete blood count and biochemistry) and urine analysis; no lesions in the urinary tract (especially in kidneys) visible on ultrasound examination. Horses in this group had routine veterinary examinations regularly at 6-month intervals and were observed daily by the owners.

Control group II consisted of 11 horses with clinical kidney dysfunction (6 females and five males) between 2 and 20 years old (mean 9.4 SD 5.8), weight 480 SD 61 kg, which were patients of different clinics in Poland and were referred to the University Clinic of the Department of Internal Diseases with Clinic for Horses, Dogs and Cats. The criteria for inclusion in this group were clinical signs of primary disease accompanied by azotemia, lasting for more than 5 days. Horses in this group were diagnosed with AKI on admission and developed kidney dysfunction due to different primary causes, such as cardiovascular compromise (4 horses), action of potentially nephrotoxic drug (4 horses), endotoxemia (2 horses), rhabdomyolysis (1 horse). Additional criteria confirming the occurrence of kidney dysfunction in this group of horses were changes in urinalysis (increased sodium fractional excretion, increased gamma-glutamyl transpeptidase to creatinine ratio, proteinuria, hyper-/iso-/hyposthenuria) and/or changes in renal ultrasound parameters (apparent distinction between cortex and medulla, dilation of renal pelvis, increased echogenicity of the renal cortex, kidney enlargement, perirenal edema) [33, 36]. Horses from this group corresponded to stage 0 (3 horses), 1 (3 horses), 2 (3 horses) and 3 (2 horses) of AKI according to the Veterinary Acute Kidney Injury (VAKI) criteria [37]. The exclusion criteria were: history of kidney dysfunction and urinary track disease; presence of congenital urinary anomalies; post-renal causes of azotemia; evidence of chronic kidney disease (i.e. azotemia persisting for more than 90 days, azotemia combined with anemia, hypercalcemia and/or weight loss) [33]; elevated serum creatinine levels without signs of primary disease.

The research group consisted of 30 non-azotemic horses at risk for AKI—suspected or predisposed to the development of AKI (15 females and 15 males) aged 2–27 years (mean 14.7 SD 7.6), weight 494 SD 67 kg, which were patients of the University Clinic. This group contained horses at risk of developing AKI, including ten intensive care patients with gastrointestinal diseases (present as colic), some of which were referred for laparotomy; ten horses receiving systemic phenylbutazone p.o. at 2.2 mg/kg q12h for ten days; ten horses receiving systemic gentamicin i.v. at 6.6 mg/kg for 5 days. The drugs were administered for a number of causes, such as skin wounds, treatment of orthopedic injuries and disorders, inflammation of the upper or lower respiratory tract, etc. Horses in this group did not show systemic manifestation of the primary disease, such as endotoxemia, sepsis or systemic inflammatory response syndrome. None of the horses in this group received fluid therapy prior to blood sampling. All the horses from this group had to have serum creatinine and urea within physiological limits at the time of admission and could have had a slight elevation of serum creatinine during the study trial (no more than 25% above the baseline values) [37].

The history of all the horses was collected from the owners. All the horses underwent a full clinical examination, urinary tract ultrasound examination, blood analysis and a complete urinalysis with sediment examination. Body weight was determined with a measuring tape dedicated to horses (in the case of horses examined in the stable) or with a scale for horses (in the case of horses kept at the University Clinic). Samples from these horses have been included in previous publications; therefore, some data, i.e., the clinical assessment and basic serum and urine measurement, are duplicated (article in review).

In accordance with the Experiments on Animals Act from January 15th 2015 (Journal of Laws of the Republic of Poland, 2015, item. 266), concerning the welfare of the animals used for research or teaching purposes, the provisions shall not apply to: 1. veterinary services as defined by the Act from December 18th 2003 concerning veterinary practices (Journal of Laws from 2004, No. 11, item 95 as amended in item 3), as well as agricultural activity, raising and breeding livestock according to the Animal Welfare Act, not designed to carry out medical procedures; 2. clinical veterinary studies carried out according to Article 37ah-37ak of the Act from September 6th 2001 –Pharmaceutical Law (Journal of Laws from 2008, No. 45, item 271 as amended in item 4); 3. activity aimed at identifying animals; 4. capturing wild animals for biometric and systematic assessment; 5. veterinary procedures which do not cause pain, suffering, distress or permanent health impairment equal to or more invasive than an insertion of a needle. Hence, the study does not require the approval of the Ethics Committee. The study was performed on privately owned horses, hence the owners provided informed consent for the clinical research and publication of the results.

### Blood examination

Blood was collected from the jugular vein with a 20G needle and a 20 ml syringe into 2 ml EDTA tubes (Medan, Poland) and 10 ml tubes with a clot activator (Medan, Poland). The hematological examination was performed using the Scil Vet ABC^TM^ Animal Blood Counter (Horiba Medical, USA) with dedicated reagent ABX Vetpack (Horiba Medical, USA, catalog number SAP1210604052). The following parameters were measured: the concentration of hemoglobin (HGB), hematocrit (HT), the red blood cell count (RBC), the white blood cell count (WBC). The blood biochemistry was determined using the Olympus AU680 chemistry analyzer (Beckman Coulter, California, USA) and dedicated reagents (Beckman Coulter, California, USA). The concentration of serum creatinine (cat.no. OSR6178), urea (cat.no. OSR6234), aspartate aminotransferase (AST) (cat.no. OSR6209), gamma-glutamyl transpeptidase (GGT) (cat.no. OSR6120), alkaline phosphatase (AP) (cat.no. OSR6204), creatine kinase (CK) (cat.no. OSR6279), total protein (TP) (cat.no. OSR6232), albumin (cat.no. OSR6202), glucose (cat.no. OSR6221), sodium (Na) (cat.no. MU919400), potassium (K) (cat.no. MU919500), chlorine (Cl) (cat.no. MU919600), magnesium (Mg) (cat.no. OSR6189), calcium (Ca) (cat.no. OSR60117), phosphate (Ph) (cat.no. OSR6222) were determined.

### Examination of urine

Urine was collected into a sterile container during spontaneous urination from all horses except those with oliguria and surgical colic. In the latter horses, urine was collected aseptically via catheterization, under pharmacological sedation or general anesthesia. Urine samples were collected once from each horse: in the healthy horses—on the day of clinical trials; in horses with kidney disease—on admission to the clinic, before treatment of kidney decline and in suspected horses—this varied (prior to the surgical treatment of colic, following drug administration in horses receiving gentamicin or NSAIDs). The following urine parameters were evaluated in physicochemical examination and using refractometer, pH-meter (CP-411, Elmetron, Poland) and urine strip test (ComboStik 10M, DFI Co. Ltd., South Korea, cat.no. 2004; strip reading device: ComboStik R-700, DFI Co. Ltd., South Korea): color, transparency, specific gravity, pH, concentration of glucose, blood, acetone, urobilinogen and bilirubin. Other biochemical parameters, such as protein (cat.no. OSR6232), creatinine (cat.no. OSR6178), GGT (cat.no. OSR6120), and sodium (cat.no. MU919400), was measured using the Olympus AU680 chemistry analyzer (Beckman Coulter, California, USA) and dedicated reagents (Beckman Coulter, California, USA). The urine protein creatinine ratio (UPC) and urine GGT to creatinine ratio (GGT: Crea) were calculated. Fractional excretion of sodium (FENa) was calculated using formula: [FENa (%) = 100 x (urine Na x serum Creatinine/serum Na x urine Creatinine)]. The 2 ml full urine sample was placed in an Eppendorf tube (Eppendorf, Germany) and stored at -80°C until its transport to the Department of Biochemistry and Molecular Biology, Faculty of Veterinary Medicine, Wroclaw University of Environmental and Life Sciences, to perform ELISA test. Remaining urine was centrifuged for 10 min. at 2500x g using a MPW-250 laboratory centrifuge (MPW Med. Instruments, Warsaw, Poland) to obtain sediment. The urine sediment was evaluated under a microscope to assess the presence of cells, casts and crystals. A low speed of centrifugation prevented podocyte damage. 0.7 mL of sediment was transported to the Faculty of Chemistry, University of Wroclaw, Poland, for LC-MS-MRM analysis.

### Peptide synthesis

All the solvents and reagents were used as supplied. Fmoc amino acid derivatives and the Fmoc-Arg (Pbf)-Wang resin (0.32 mmol/g), Fmoc-Lys (Mtt)-Wang resin (0.56 mmol/g) were purchased from Novabiochem (Darmstadt, Germany). N-[(Dimethylamino)-1H-1,2,3-triazolo-[4,5-b] pyridin-1-ylmethylene]-N-methylmethanaminium hexafluorophosphate N-oxide (HATU) and trifluoroacetic acid (TFA) were obtained from IrisBiotech. Solvents for peptide synthesis (N,N-dimethylformamide (DMF), dichloromethane (DCM), and (N-ethyldiisopropylamine (DIEA) and tetraethylammonium bicarbonate (TEAB) and *N*,*N*,*N*-triethylamine, 1,2-dithiotreitol (DTT), NP40 (Tergitol) and iodoacetamide were obtained from Sigma Aldrich (Saint Luis, MO, USA); triisopropylsilane (TIS) was purchased from Fluka (Bucharest, Romania). Amicon® Ultra Centrifugal Filters were purchased from Merck (Darmstadt, Germany).

Model peptides were synthesized on the Fmoc-Lys(Mtt)-Wang and Fmoc-Arg(Pbf)-Wang manually in polypropylene syringe reactors (Intavis AG, Tübingen, Germany) equipped with polyethylene filters, according to a standard Fmoc (9-fluorenylmethoxycarbonyl) solid-phase synthesis procedure [38].

#### Purification

The synthesized peptide was purified with the use of the analytical HPLC Thermo Separation system and UV detection (210 nm) with a YMC-Pack RP C18 column (4.6 × 250 mm, 5 μm), and a gradient elution of 0–40% of B in A (A = 0.1% TFA in water; B = 0.1% TFA in acetonitrile/H2O, 4: 1) over 30 min (flow rate 1 ml/min). The main fraction, corresponding to the peptide, was collected, lyophilized and confirmed in the MS/MS experiment.

#### Mass spectrometry

All the ESI-MS experiments were performed on a micrOTOF-Q mass spectrometer (Bruker Daltonics, Bremen, Germany) equipped with a standard ESI source. The instruments were used in the positive-ion mode and calibrated with the Tunemix™ mixture (Agilent Technologies, Palo Alto, CA, USA). The mass accuracy was higher than 5 ppm. The analyte solutions (70 μl) were introduced at a flow rate of 3 μL/min. The instrument parameters were as follows: the scan range of the micrOTOF-Q MS was 50–1600 m/z; nitrogen was used as the drying gas; the flow rate was 4.0 L/min, the temperature was 200°C; the potential between the spray needle and the orifice was 4.2 kV.

#### CID

The singly ([M+H]^+^) and doubly protonated ([M+2H]^2+^) precursor ions were selected on the quadrupole and subsequently fragmented in a hexapole collision cell. Argon was used as the collision gas. The obtained fragments were registered as an MS/MS (tandem mass spectrometry) spectra. The collision energy (10–30 V) was optimized for the best fragmentation. Bruker Compass DataAnalysis 4.0 software was used for MS spectral analysis.

#### Urine sample preparation

The pellet of the collected urine samples was re-suspended in 1 mL of 0.1 M TEAB buffer containing NP40 (Tergitol) at the concentration of 0.01% and sonicated for 15 min. The sample was transferred into the Amicon® Ultra Centrifugal Filter and centrifuged at 4000 rpm for 15 min. The sample was then diluted with a 0.1 M TEAB solution and centrifuged again (washing out the impurities with low molecular weight). In the next step, 100 μL of 0.2 M DTT was added, the sample was incubated for 1 h at 30°C and centrifuged at 4000 rpm for 15 min. DTT was removed by means of washing the sample with a 0.1 M TEAB buffer and centrifugation. The groups were blocked by the reaction with 100 μL of 0.1 M iodoacetamide (CAM, 1 h, room temperature). Excess CAM was removed by washing the sample with 0.1 M TEAB and by centrifugation. The obtained supernatant was placed in an Eppendorf tube. Then, 50 μg of trypsin was added in 200 μL of 0.1 M TEAB, and the sample was incubated at 37°C overnight. After digestion, 20 μL of formic acid was added and the sample was lyophilized. The dry solid was dissolved in 5% MeCN/H_2_O and used for the LC-MS-MRM analysis.

#### Liquid Chromatography-Mass Spectrometry (LC-MS) analysis in Multiple Reaction Monitoring (MRM) mode

LC-MRM experiments were performed on an Agilent Technologies 6470 Triple Quad LC/MS device (Palo Alto, CA, USA), with an Agilent Technologies 1290 Infinity II system equipped with a 3.6 μm bead diameter Aeris Peptide XB-C18 column (50 mm × 2.1 mm), equilibrated at 24°C and on the LCMS-8050 Shimadzu apparatus, with a UHPLC Nexera X2 system, equipped with a 3.6 μm bead diameter Aeris Peptide XB-C18 column (50 mm × 2.1 mm), equilibrated at room temperature. The LC system was operated with a mobile phase, consisting of solvent A: 0.1% formic acid in H_2_O and solvent B: 0.1% formic acid in MeCN. The gradient conditions (B %) were from 0 to 65% B within 14 min. The flow rate was 0.4 mL/min and the injection volume was 2 μL.

The MRM method was developed manually, and the following transitions were chosen: 833.0→242.1 (b2), 833.0→970.6 (y8), 833.0→825.9 (b14^2+^) for the peptide with the ^169^H-LQTLEIPFHEIVTK-OH^182^ sequence and 653.1→630.3 (y8) and 635.1→308.2 ([TPP+H]^+^) for its H-LQTLEIPFHEIVTK(TPP)-OH derivative.

#### Peptide derivatization in solution with 2,4,6-triphenypyrilium salt

The purified peptide with the ^169^H-LQTLEIPFHEIVTK-OH^182^ sequence (0.2 mg) was dissolved in 0.5 mL of DMF containing 0.05 mg of 2,4,6-triphenylpyrilium tetrafluoroborate and 19 μL of *N*,*N*,*N*-triethylamine. The mixture was incubated at 60°C for 1 h, and the solution was evaporated under a nitrogen stream. The final product was dissolved in 5% MeCN/H_2_O mixture (v: v), lyophilized and used for MS/MS analysis and the development of the MRM method.

#### Urine tryptic digest derivatization with 2,4,6-triphenypyrilium salt

The lyophilized tryptic urine hydrolates were dissolved in 0.5 mL of a DMF solution containing 0.25 mg of 2,4,6-triphenylpyrilium tetrafluoroborate and 95 μL of *N*,*N*,*N*-triethylamine. The mixture was incubated at 60°C for 1 h and the solution was evaporated under a nitrogen stream. The final product was dissolved in a 5% MeCN/H_2_O mixture (v: v) and lyophilized.

### Quantitative podocin evaluation–ELISA test

Full horse urine was used for the quantitative analysis of the podocin content. After thawing, the samples were mixed thoroughly and centrifuged briefly at a speed of 4000 x g for 5 minutes. Undiluted urine was used to measure podocin (pg/ml) levels using commercially available validated species-specific ELISA (MyBioSource, USA, cat.no. MBS023791) according to the manufacturer’s manuals. The plates were washed with an Automatic Plate Washer ELx50 (BioTek, USA) and absorbance was assessed with Multiscan EX (Thermo, USA). For each measurement, a 620 nm background absorbance was subtracted from the 450 nm absorbance, and a 4-parameter curve fitting was employed to calculate the analyte concentration. All measurements were performed in duplicates. In order to enable comparison of results to literature data, the unit was converted from pg/ml to ng/ml.

### Comparison of the results obtained with the use of the qualitative and quantitative method

To compare the results obtained with the use of two different methods, the maintained results were unified. The results obtained in the qualitative method were divided into: negative (-): for no presence of podocin and positive (+): for detection of trypsin podocin peptide fragments. Results from the quantitative method were divided into groups, depending on the amount of podocin in a given sample, based on the cut-off value and scale adopted by the authors: negative (-) for value lower than 0.81 ng/ml, equivocal (-/+) for value equal to 0.81 ng/ml and positive (+) for value higher than 0.81 ng/ml.

### Statistical analysis

The normal distribution of data was analyzed with the Shapiro-Wilk test. One way ANOVA with Fisher’s LSD post-hoc test was used to compare normally distributed variables. Normally distributed variables were presented as average ± standard deviation (SD), abnormally distributed variables were presented as median and quartile. The differences between the groups in the quantitative variables were evaluated with the Kruskal-Wallis test, using the post-hoc Conover pairwise comparison with the Benjamini–Hochberg procedure, or Fisher's exact test for qualitative variables. Additionally, multivariate linear regression was used to assess the difference in variables between groups, with adjustment for age and sex. The correlation between variables was evaluated using the Spearman correlation coefficient. The Kruskal-Wallis test was used to measure the effect of the variables on the presence of podocin in urine. A Receiver Operating Characteristic (ROC) was performed for quantitative test results to calculate the diagnostic sensitivity and specificity, positive (PPV) and negative predictive value (NPV) of podocin to detect kidney injury. Cohen’s Kappa was used to assess the compliance between the quantitative and qualitative podocin test. The tests were considered statistically significant if p<0.05. All analyses were performed using R for Windows software (version 3.6.1).

## Results

### Study population

The data of the measured blood and urine parameters in healthy horses, horses with clinical kidney dysfunction and horses at risk for AKI are presented in Tables 1 and 2.

**Table 1:** Hematological and biochemical blood results of healthy horses, horses with clinical kidney dysfunction (horses with AKI) and horses at risk for acute kidney injury.

Parameter	Healthy horses (n = 30)			Horses with AKI (n = 10)			Horses at risk for AKI (n = 30)			P Value
	Q1	Me	Q3	Q1	Me	Q3	Q1	Me	Q3	
**HGB (mmol/l)**	7.0	7.4	7.7	6.5	7.2	8.8	6.5	7.1	8.4	NS
**HCT (l/l)**	0.34	0.37	0.38	0.30	0.35	0.42	0.31	0.37	0.43	NS
**RBC (10**^**12**^**/l)**	7.5	8.0	8.5	5.9	7.6	9.0	5.9	7.2	8.3	NS
**WBC (10**^**9**^**/l)**	5.9	6.8	8.8	8.0	9.3	15.6	7.2	7.9	9.1	NS
**Urea (mmol/l)**	4.6	5.7	6.3	10.6	12.3	23.9	5.3	5.7	6.4	<0.0001*^#^
**Crea (umol/l)**	93.0	120.0	137.0	228.0	305.0	440.2	103.8	129.0	136.9	<0.0001*^#^
**AST (U/l)**	264.0	283.0	321.0	244.0	416.7	501.5	247.0	300.5	342.7	NS
**GGT (U/l)**	10.0	12.0	16.0	16.5	23.5	44.0	9.0	14.0	21.6	0.04*
**AP (U/l)**	172.0	201.0	262.0	185.5	204.0	375.0	140.6	184.5	224.3	NS
**CK (U/l)**	196.0	227.0	311.0	186.5	221.0	313.5	182.0	224.0	285.0	NS
**Total protein (g/l)**	59.0	63.0	70.0	54.7	62.0	70.0	60.0	61.0	64.2	NS
**Albumin (g/l)**	31.0	32.0	34.0	20.4	28.0	36.5	24.0	30.0	33.8	NS
**Glucose (mmol/l)**	3.8	4.7	5.3	4.6	5.6	6.4	4.5	4.9	5.5	0.03*
**Sodium (mmol/l)**	134.0	135.8	136.8	129.1	134.0	137.5	131.0	133.9	136.8	NS
**Potassium (mmol/l)**	3.5	4.0	4.1	2.3	2.8	4.1	3.5	4.0	4.4	NS
**Chlorine (mmol/l)**	98.4	99.0	100.4	86.5	96.0	99.7	98.8	100.0	101.7	0.02^#^
**Magnesium (mmol/l)**	0.7	0.7	0.78	0.64	0.73	0.8	0.66	0.71	0.74	NS
**Calcium (mmol/l)**	2.9	3.1	3.2	2.2	3.2	3.6	2.6	2.9	3.1	NS
**Phosphate (mmol/l)**	0.7	0.9	1.1	0.96	1.1	1.5	0.8	1.06	1.3	NS

*Note*: AKI–acute kidney injury; Q1 –quartile 1; Me–median; Q3 –quartile 3; HGB—hemoglobin; HCT—hematocrit; RBC–red blood cell count; WBC–white blood cell count; AST–aspartate aminotransferase; GGT–gamma-glutamyl transpeptidase; AP–alkaline phosphatase; CK–creatine kinase; NS–statistically insignificant.

*—p<0.05 for healthy horses and AKI horses.

^—p<0.05 for healthy horses and horses at risk for AKI

^#^—p<0.05 for AKI horses and horses at risk for AKI.

**Table 2:** Urine biochemistry results of healthy horses, horses with clinical kidney dysfunction (horses with AKI) and horses at risk for acute kidney injury.

Parameter	Healthy horses (n = 30)			Horses with AKI (n = 10)			Horses at risk for AKI (n = 30)			P Value
	Q1	Me	Q3	Q1	Me	Q3	Q1	Me	Q3	
**pH**	8.0	8.0	8.4	5.45	6	7.95	7.8	8.0	8.5	0.001*^#^
**Specific gravity (g/l)**	1.021	1.031	1.042	1.011	1.013	1.0225	1.02	1.033	1.036	NS
**Protein (g/l)**	0.03	0.07	0.10	0.375	0.69	1.24	0.13	0.205	0.315	<0.0001*^^^
**Creatinine (mmol/l)**	13.9	20.9	33.8	3.5	9.1	15.0	12.7	17.1	22.5	NS
**UPC**	0.014	0.023	0.04	0.35	0.65	3.1	0.075	0.095	0.16	<0.0001*^^^
**GGT**	18.0	21.0	20.5	22.0	20.5	23.0	19.3	25	32	NS
**GGT: Crea (IU/mmol)**	0.74	1.07	1.52	1.62	4.55	7.19	1.22	1.44	1.97	<0.05*^#^
**Sodium (mmol/l)**	15.6	20.2	24.3	26.9	28.9	56.6	13.5	23.8	40.7	NS
**FENa (%)**	0.06	0.08	0.12	0.44	1.95	5.80	0.07	0.13	0.17	<0.005*^#^

*Note*: AKI–acute kidney injury; Q1 –quartile 1; Me–median; Q3 –quartile 3; UPC—urine protein creatinine ratio; GGT–Gamma-glutamyl transferase; Crea–creatinine; FENa—Fractional sodium excretion; NS–statistically insignificant.

*—p<0.05 for healthy horses and AKI horses.

^—p<0.05 for healthy horses and horses at risk for AKI

^#^—p<0.05 for AKI horses and horses at risk for AKI.

There were no statistically significant differences in age between the various horse groups. Healthy horses were heavier than the horses in the AKI group and that at risk for AKI. Healthy horses and horses at risk for AKI had significantly lower blood creatinine and urea than horses with AKI. Also in the horses with AKI, the serum GGT and glucose were significantly higher than in healthy horses. Serum chloride concentration was significantly higher in the group of suspected horses compared to the horses with AKI. Other blood parameters did not differ significantly between the groups, despite an increased concentration of some of them in individual animals and the highest median in horses with AKI (like WBC and AST). Horses with AKI had a significantly lower urine pH than the remaining horses. The average urine specific gravity was lower compared to the remaining two groups, but this difference was not statistically significant. Five horses from the AKI group and four from the at risk group had an increased urine protein concentration. Horses with a clinical form of kidney disease had the highest concentration of protein in urine. Total urine protein concentration in horses at risk for AKI, as well as those with AKI, was statistically higher than in healthy horses. Five AKI horses and two at risk horses had elevated UPC. The UPC value was significantly lower in healthy horses compared to other groups. Of all tested horses, only five horses with AKI had an elevated GGT: Crea ratio. In this group, this ratio was significantly higher than in the other groups. Only six horses with AKI and one horse from the group at risk for AKI treated with NSAIDs had a higher than normal fractional sodium excretion. In the AKI group, this value was significantly higher than in the other animals.

None of the horses from the healthy group developed kidney dysfunction within the 6 month period following the study. Two horses from the group of horses suspected of AKI had slightly elevated serum creatinine concentration after the end of the study- one horse receiving NSAIDs and one colic horse. The latter also had an increased urinary GGT.

### Qualitative podocin results

**Fig 1:**
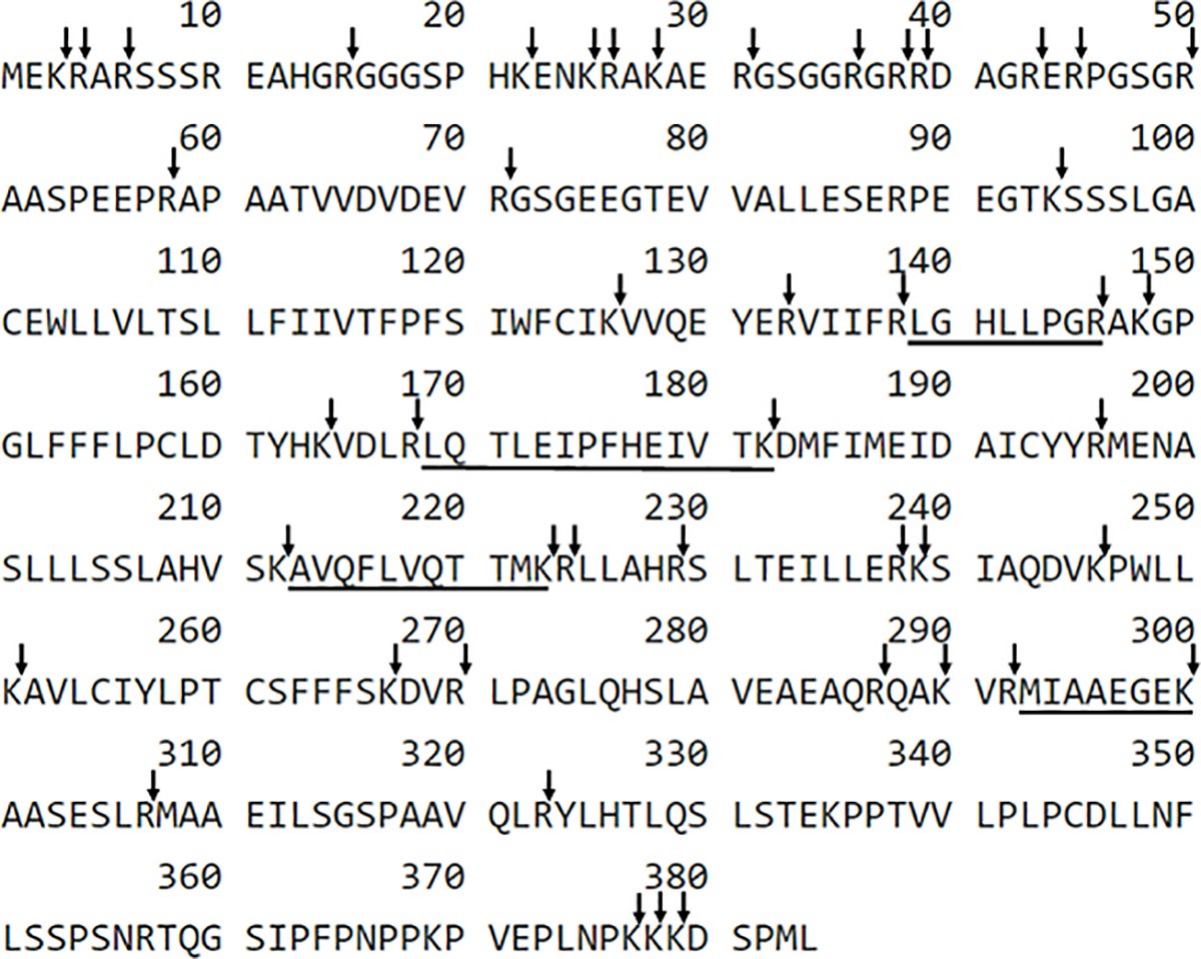
The sequence of horse podocin with marked trypsin cleavage sites determined according to the UniProt databases (entry name F6WST7_HORSE). The selected sequences are underlined.

The tryptic equine podocin peptides with ^139^H-LGHLLPGR-OH^146^, ^169^H-LQTLEIPFHEIVTK-OH^182^, ^213^H-AVQFLVQTTMK-OH^223^, ^293^H-MIAAEGEK-OH^300^ sequences were chosen as a potential podocin biomarker (Fig 1).

**Fig 2:**
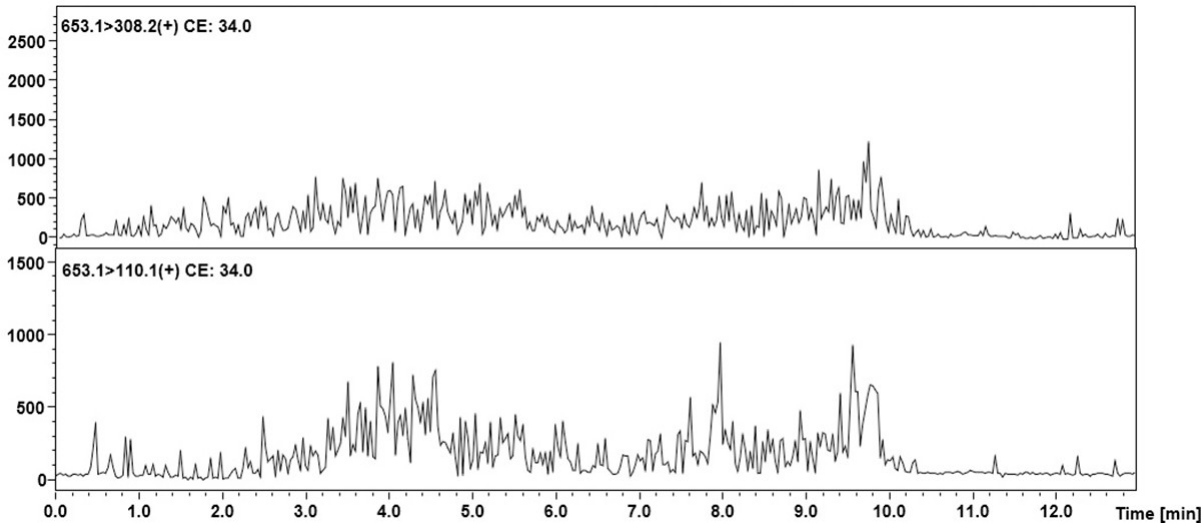
MRM analysis of the urine sediment tryptic digest sample which does not contain podocin. The peaks characteristic for the transitions of the analyzed tryptic podocin peptide were not identified.

**Fig 3:**
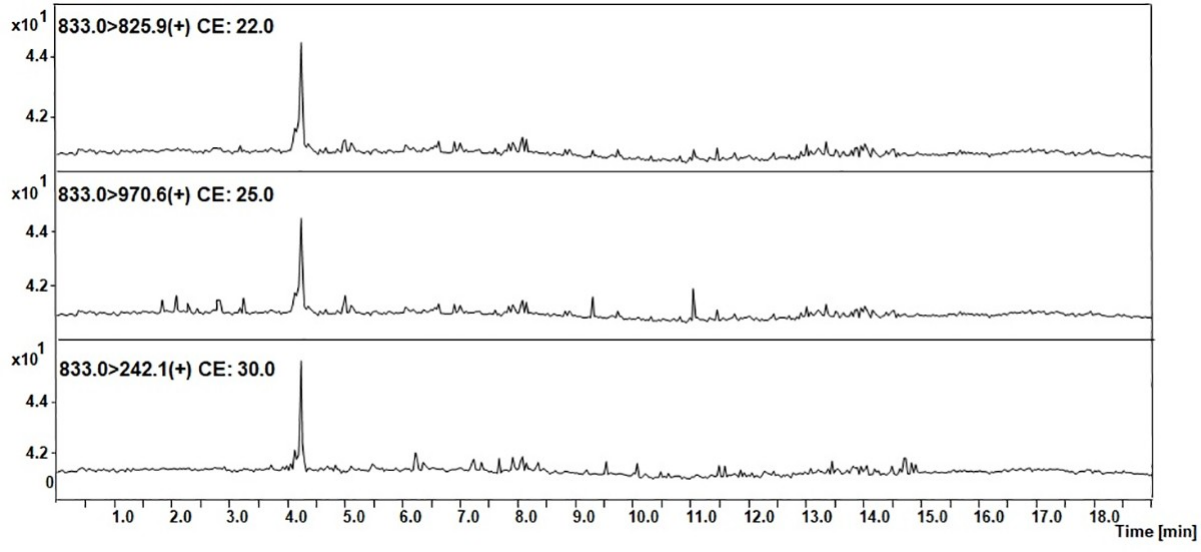
MRM chromatograms representing transitions corresponding to the model peptide with the ^169^H-LQTLEIPFHEIVTK-OH^182^ sequence identified in the tryptic digest of an equine urine sediment sample from animal with diagnosed AKI.

Additionally, ^139^H-LGHLLPGR-OH^146^, ^169^H-LQTLEIPFHEIVTK-OH^182^, ^213^H-AVQFLVQTTMK-OH^223^ sequences are characteristic both of human and equine podocin. Detection sensitivity for all of the obtained compounds was analyzed using LC-MS/MS and LC-MS-MRM methods in order to find the best peptide which may be present in trace amount in urine samples for the identification of equine podocin. The obtained data confirmed that the peptide with the ^169^H-LQTLEIPFHEIVTK-OH^182^ sequence was characterized at the subfemtomolar level of detection (1×10^−15^ mole). Other sequences were identified at the level of 10^−14^ mole. The performed study allowed to choose the peptide with the ^169^H-LQTLEIPFHEIVTK-OH^182^ sequence as a model for the identification of podocin in equine urine samples. For the selected peptide, the MRM method was optimized as the most intensive signal on the obtained mass spectra using the [M+2H]^2+^ (m/z 833.0) ion. The following MRM transitions 833.0→242.1 (b_2_), 833.0→970.6 (y_8_), 833.0→825.9 (b_14_^2+^) were selected and used in the investigation of equine podocin in the urine sediment samples. The retention time of the chosen peptide under chromatographic separation conditions presented in the materials and methods section was 4.2 min. The samples were prepared according to the procedure described in the “materials and methods” section. The results concerning the sample obtained from the healthy animals and animals diagnosed with AKI are presented in Figs 2 and 3.

The obtained MRM chromatograms for the urine sediment samples from the animals with diagnosed AKI (Fig 3) show peaks corresponding to the transition characteristic for the selected equine tryptic podocin peptide with the ^169^H-LQTLEIPFHEIVTK-OH^182^ sequence with the confirmed retention time. Based on the obtained MRM chromatograms, it can be assumed that podocin was found in the analyzed samples.

**Fig 4:**
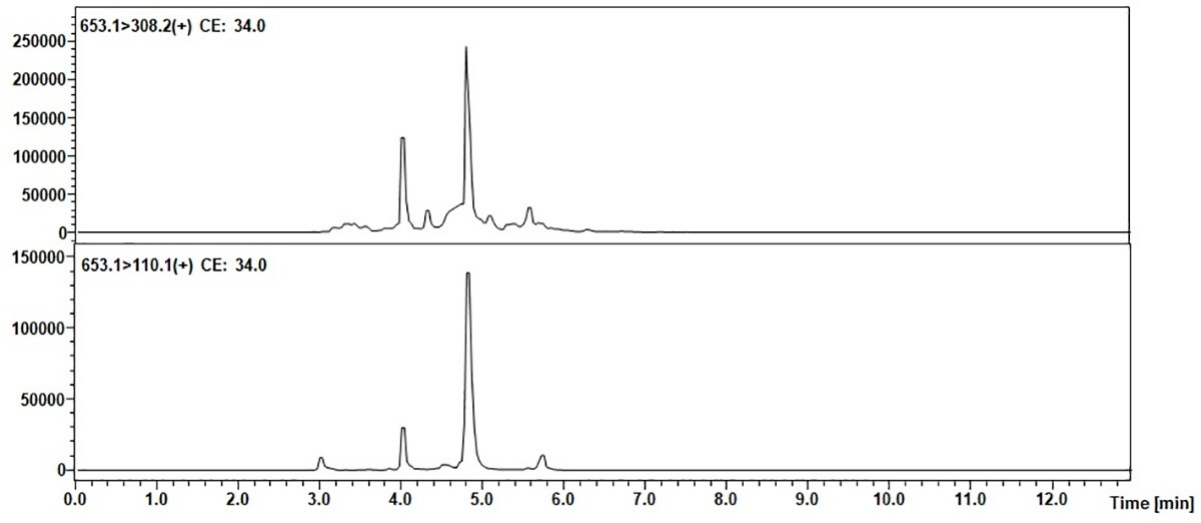
MRM chromatograms representing transitions corresponding to the model peptide with the H-LQTLEIPFHEIVTK(TPP)-OH sequence identified in the tryptic digest of an equine urine sediment sample from animal with diagnosed AKI.

Mass spectrometric analysis of trace amounts of peptides may be problematic due to insufficient ionization efficiency resulting in limited sensitivity. One of the possible ways to overcome this problem is to apply ionization enhancers [39]. Therefore, the authors chose to apply their own strategy of the peptide modification using the 2,4,6-triphenylpyrilium salt as an ionization marker [40]. The model peptide with the ^169^H-LQTLEIPFHEIVTK-OH^182^ sequence was modified according to the procedure described in the materials and methods section. The obtained H-LQTLEIPFHEIVTK(TPP^+^)-OH derivative was used to develop the MRM method. The MRM method was optimized for the selected TPP-peptide derivative using the [M+2H]^3+^ (m/z 653.1) ion, as the most intensive signal on the obtained mass spectra. The following MRM transitions 653.1→630.3 (y_8_^2+^) and 635.1→308.2 ([TPP+H]^+^) were selected and used in the analysis of the presence of equine podocin in the collected urine sediment samples. The retention time of the chosen peptide under chromatographic separation conditions presented in the materials and methods section was 4.8 min. The obtained results for the analyzed samples are presented below (Fig 4).

The obtained MRM data (Fig 4) for the urine sediment samples derivatized by using 2,4,6-triphenylpyrilium salt as the ionization marker showed peaks corresponding to the transition characteristic for the selected equine tryptic podocin peptide with the H-LQTLEIPFHEIVTK(TPP^+^)-OH sequence, additionally confirmed by the retention time. Based on the obtained MRM chromatograms, it can be assumed that podocin was found in the analyzed samples.

In summary, signals that may indicate trypsin fragments of podocin were found in three healthy horses, all the animals diagnosed with kidney disease (11 horses) and half of the animals at risk for AKI (15 horses– 5 horses that received NSAID, 6 colic horses, 4 horses that receiving gentamicin). Only in the case of one of the aforementioned horses at risk for AKI (receiving NSAIDs), in whom serum creatinine increased, the presence of podocin was confirmed.

The Kruskal-Wallis analysis showed that higher serum creatinine and urea levels were associated with the presence of podocin in the urine sample. Also, the presence of proteinuria, increased UPC, GGT: Crea ratio, and FENa were associated with the presence of podocin. Lower urine pH was found to relate to the presence of podocin in the tested urine sample.

### Quantitative podocin results

**Fig 5:**
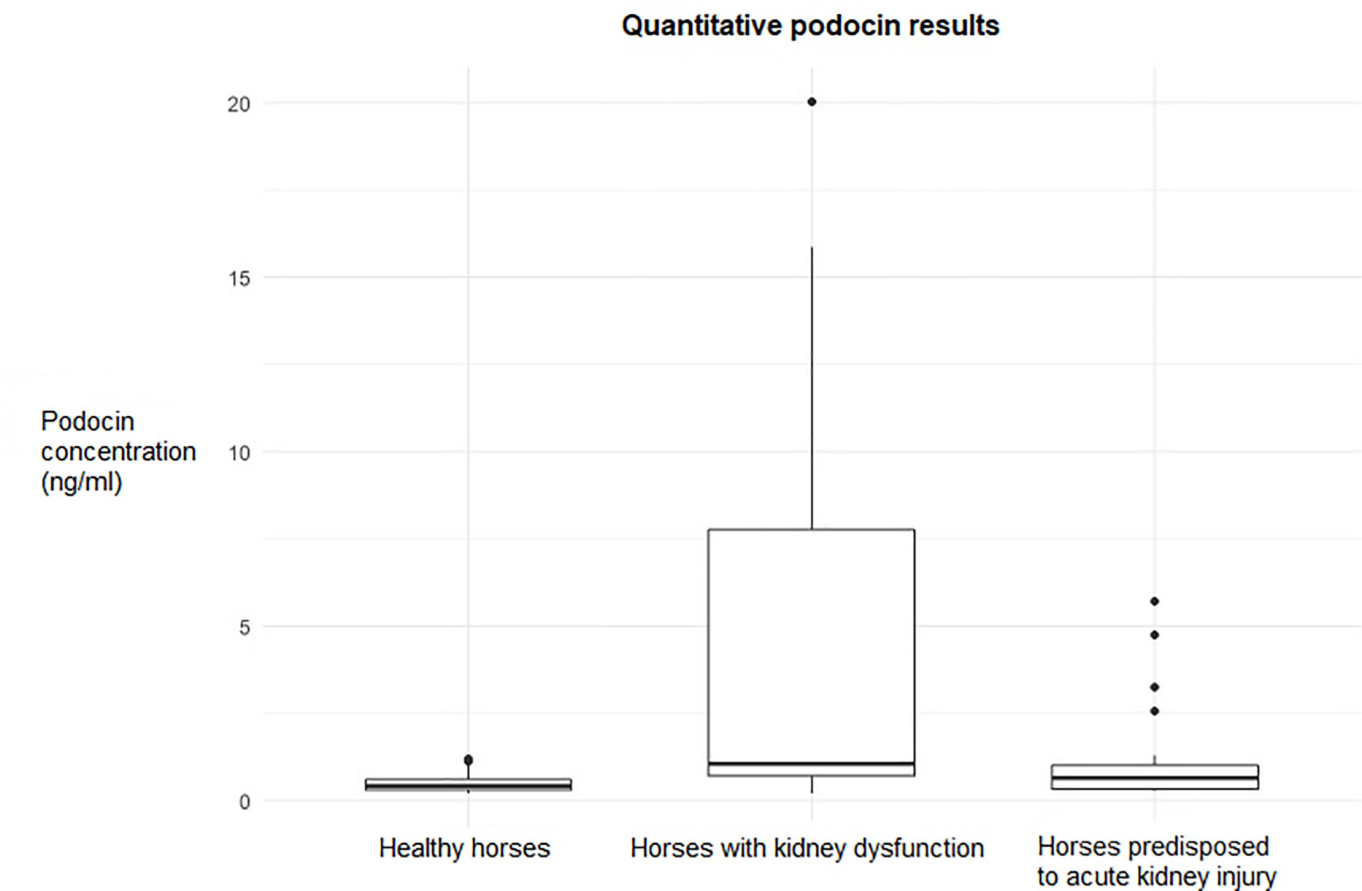
Graph showing results of the quantitative podocin ELISA test in healthy horses, horses with clinical kidney dysfunction and horses at risk for acute kidney injury.

The results of the ELISA podocin test for the examined horses are presented in Table 3 and Fig 5.

**Table 3:** Results of the podocin concentration based on the quantitative ELISA test in healthy horses, horses with clinical kidney dysfunction (horses with AKI) and horses at risk for acute kidney injury.

	Healthy horses		Horses with AKI		Horses at risk for AKI		
	Min.–max.	Me (Q1 –Q3)	Min.–max.	Me (Q1 –Q3)	Min.–max.	Me (Q1 –Q3)	P Value
**Podocin (ng/ml)**	0.19–1.2	0.428 (0.312–0.618)	0.19–20.0	1.053 (0.714–7.775)	0.29–5.71	0.658 (0.317–1.004)	0.01*

*Note*: AKI–acute kidney injury; Q1 –quartile 1; Me–median; Q3 –quartile 3

*—p<0.05 for healthy horses and AKI horses.

In the AKI horse group, the mean values of podocin concentration for the subsequent stages of AKI were: 4.3 SD 5.9 ng/ml for VAKI stage 0, 0.4 SD 0.2 ng/ml for stage 1, 2.4 SD 1.8 for stage 2 and 17.9 SD 2.9 for VAKI stage 3. AKI horses had a statistically significantly higher podocin level than the rest on the examined animals. There were no statistically significant interactions between the podocin concentration and age in the study group. However, in the group of sick animals, a trend of decreasing podocin levels with age was observed (p = 0.06254). In this group, a trend of increased concentrations of podocin in males compared to females was also observed (p = 0.063). Spearman’s correlation indicated a low positive correlation between the podocin level and UPC (r = 0.03, p = 0.013) in all the examined horses, as well as between podocin and FENa (r = 0.3, p = 0.03). No significant correlation was observed between the podocin concentration and other clinical, blood and urine parameters. When each study group was analyzed separately, no significant correlations were found between any blood and urine parameters tested and podocin.

**Fig 6:**
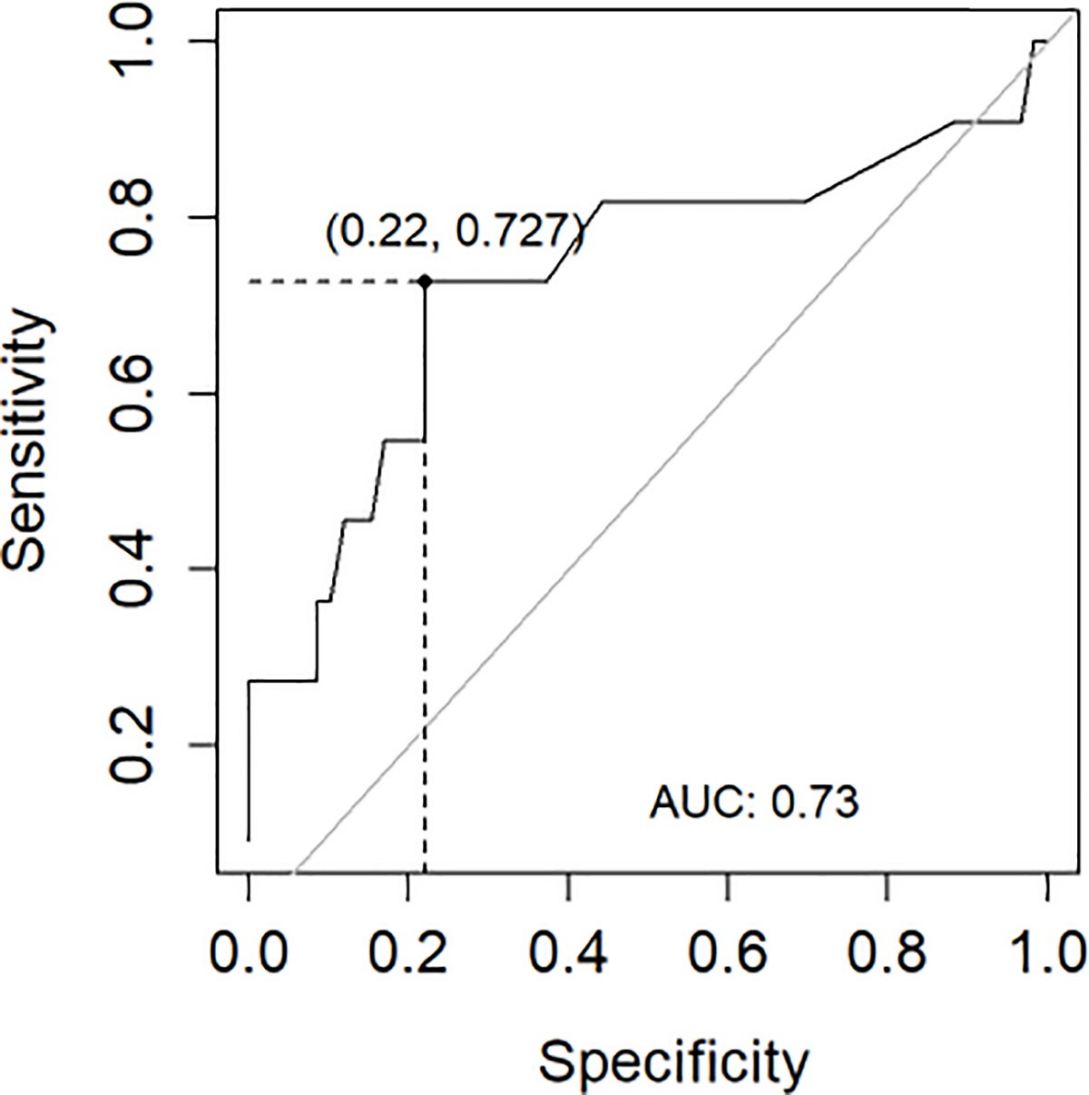
The ROC analysis curve, showing sensitivity and specificity for podocin ELISA test.

Table 4 and Fig 6 contains the results of the ROC analysis of the concentration of podocin to detect kidney disease.

**Table 4:** The ROC analysis of podocin relative to the kidney disease in horses.

Cut-off value	Sensitivity	Specificity	Positive predictive value	Negative predictive value	AUC*
0.81	0.73	0.78	0.38	0.94	0.73

Note

*AUC—area under the ROC.

The best cut-off value for podocin was 0.81. The podocin ELISA test proved to be more effective in detecting negative results than positive ones. The test's ability to distinguish between normal and abnormal results was fair and was 0.73.

Podocin values above the cut-off value was observed in 4/30 healthy horses, 7/11 horses with kidney dysfunction and 9/30 horses at risk for AKI. In the latter group, two individuals mentioned earlier with increased creatinine concentration, had podocin concentration below cut-off value.

### Comparison of qualitative and quantitative methods

Quantitative and qualitative result categories are compared in Tables 5 and 6.

**Table 5:** Total number of positive, negative and uncertain results in the quantitative (ELISA) and qualitative (LC-MS-MRM) podocin evaluation in all the examined horses.

	Qualitative method (LC-MS-MRM)		Quantitative method (ELISA)		
Result	Negative	Positive	Negative	Equivocal	Positive
Number	42	29	50	1	20

**Table 6:** Comparison of compatibility of obtained result categories between the qualitative and quantitative method.

		Qualitative method (LC-MS-MRM)		
		Negative	Positive	Total
**Quantitative method (ELISA)**	Negative	41	9	50
	Equivocal	0	1	1
	Positive	1	19	20
	Total	42	29	71

*Note*: For ELISA test: negative < 0.81, equivocal = 0.81, positive > 0.81.

There were 41 corresponding negative results in both tests (57,7% of all results) and 19 positive results (26.8% of all results). The overall compatibility of the results was 84.5%. The LC-MS-MRM qualitative method was more likely to be positive than the ELISA test. The conformity assessment of the results of both tests using the Cohen Kappa test showed compatibility with the results at 0.69. According to the value of the Kappa level of agreement, there was substantial compatibility between the tests.

## Discussion

The presented study is the first to evaluate the presence and concentration of podocin in healthy horses, as well as horses with clinical renal dysfunction. It is also the first study performed on horses at risk for AKI due to gastrointestinal disease, as well as in patients receiving potentially nephrotoxic drugs, to see how those factors affect kidneys. The study focused on the qualitative analysis of tryptic podocin peptides and the quantitative determination of podocin in urine samples in horses as a potential biomarker of podocytes and also assessed the correlation of selected serum and urine results. Additionally, in comparison to the LC-MS-MRM podocin analysis in canine and feline urine samples, this study included a novel application of ionization enhancers in the form of 2,4,6-triphenylpyridinium salts which increased the ionization efficiency of peptides allowing for their ultrasensitive analysis even at a low attomolar level [39, 40].

Due to the fact that there is no described method to detect podocin in horses, the authors evaluated two different methods for podocin detection and compared them. Currently, the application of the LC-MS-MRM method, which is considered highly sensitive, may result in an earlier diagnosis of glomerular pathologies in comparison to the other commonly used methods. The LC-MS-MRM method was proposed as a potential tool in the investigation of podocyturia in urine sediments from patients with pre-eclampsia [6]. In that study, the tryptic hydrolysates of collected urine pellets were analyzed using LC-MS-MRM with the addition of an isotopically labeled peptide with the ^39^H-QEAGPEPSGSGR-OH^50^ sequence as an internal standard. The obtained data suggest the applicability of the proposed solution, and the results were in agreement with the immunological tests. Additionally, Simon and co-workers [16] proposed a new approach for the quantification of podocin in the soluble fraction rather than in the urine sediment. In that study, the podocin tryptic peptide with the ^59^H-APAATVVDVDEVR-OH^71^ sequence was chosen for protein quantification using LC-MS-MRM. Podocin may be a potential biomarker of some diseases not only in humans but also in animal urine samples including dogs, cats or horses. Therefore, there is a strong need for the development of a methodology allowing animal urine sample preparation and the identification of potent tryptic podocin fragments. The LC-MS/MS based approach has several advantages: (1) it does not require the generation of antibodies, (2) it can be multiplexed for the simultaneous measurement of multiple proteins, (3) it has the potential to be readily applied across species if the quantified peptides are conserved [16]. The following tryptic peptides sequences containing C-terminal arginine or lysine residue ^139^H-LGHLLPGR-OH^146^, ^169^H-LQTLEIPFHEIVTK-OH^182^, ^213^H-AVQFLVQTTMK-OH^223^, ^293^H-MIAAEGEK-OH^300^ which may influence the ionization efficiency during LC-MS-MRM analysis, were chosen to analyze the possibility of the application of the LC-MS-MRM method in the investigation of podocin in the equine urine samples. Finally, the authors focused their attention on a tryptic peptide with the ^169^H-LQTLEIPFHEIVTK-OH^182^ sequence because it is located outside the transmembrane region and gives intensive signals during the MS/MS experiment corresponding to the b_2_, y_8,_ and b_14_^2+^ fragment ions. Additionally, the ^169^H-LQTLEIPFHEIVTK-OH^182^ sequence meets the peptide selection criteria described by Mohammed and co-workers [41]. For the selected peptide, the MRM method was optimized for the [M+2H]^2+^ (m/z 835.0) ion, as the most intensive signal on the obtained mass spectra. The following transitions: 835.0→242.1 (b_2_), 835.0→970.6 (y_8_), 835.0→825.9 (b_14_^2+^) were selected and used to investigate podocin in the equine urine sediment samples. A tryptic peptide with the ^169^H-LQTLEIPFHEIVTK-OH^182^ sequence is also characteristic for human and equine podocin. Positive results were found in three clinically healthy horses with the use of the LC-MS-MRM method. This group of horses did not show any other changes in blood and urine tests, nor did they develop a clinical form of the disease within six months of after the study. This suggests that, similarly to people, horses may have physiological podocyturia. This phenomenon has been described in a few studies. In the study of Simon *et al*. 2014 conducted on human urine samples, podocin was detected in 13 urinary samples from healthy donors [16]. In the study by Perez-Hemandez *et al*. in 2016, urine sediments of 10% of healthy controls grew podocytes in culture [2]. Also, in the studies of Kelder *et al*. 2012 on women it has been shown that there is a loss of podocytes in the urine [9]. Podocyturia has also been shown to be higher in more concentrated urine [42]. These three healthy horses also showed the highest urine specific gravity, but statistical analysis showed no correlation between the urine specific gravity and the presence of podocin. Using the LC-MS-MRM method, the podocytes have been found in all horses with clinical kidney dysfunction. Although glomerular diseases in horses are not described as often as in cats, it can be suspected that their pathology may be more common, and according to the obtained results, damage to the tubules may be accompanied by podocyturia in many cases [36]. The presence of podocyturia may suggest an active process of glomerular injury. The presented results may indicate that the LC-MS-MRM method can be used to confirm active, progressive process of kidney injury in patients with azotemia. The challenge for the future will be determining whether the method is capable of distinguishing between an active and inactive process in horses with kidney dysfunction or disease, which is important for the therapeutic strategy. Based on the obtained results, it can also be concluded that selected tryptic peptides of podocin are detectable in horses and can be successfully used to diagnose podocyturia. The obtained results showed a relationship between the presence of podocin in the urine and an increased urea and creatinine in blood serum, as well as an increase urine protein, UPC, the ratio of GGT: Crea and an increase in FENa. These results are consistent with those obtained in humans, were a significant positive correlation was found between podocin and blood urea, as well as podocin and serum creatinine and podocin and urine protein [2, 23, 43].

The presence of podocin was also detected in half of the horsesat risk for AKI, both in horses receiving potentially nephrotoxic drugs and those with gastrointestinal disorders. It is interesting that positive results were obtained in so many horses from this group. This may mean the mentioned factor led to a detachment of podocytes from the glomerular basement membrane or contributed to glomerular damage [8, 9, 42, 44]. In the presented study podocyturia was detected in 6 colic horses. Horses with gastrointestinal disorders are exposed to hemodynamic changes that may cause renal ischemia, patchy tubular epithelial damage or even renal cortical necrosis and lead to AKI [26, 27]. The decrease in renal perfusion may be compounded by tubular cast accumulation and intratubular obstruction [45, 46]. Also, the release of endotoxins may affect the reticuloendothelial system, damage the endothelium and promote platelet adherence and release of vasoactive substances, which may cause a decrease in the glomerular filtration rate [47]. In addition, colic horses may suffer from hyperglycemia [48], which activates inflammatory cell death pathways. Considering that colic horses are exposed to oxidative stress [49] and increased intracellular reactive oxygen species, this may all together contribute to podocyte injury [50, 51]. In colic horses, podocyturia may also be the result of endothelial dysfunction caused by a systemic inflammatory response [9]. The lack of detection of podocin in the only horse in this group which had a slightly elevated creatinine level may indicate another reason for its increase, not related to the glomerulus. The routine use of nephrotoxic drugs such as non-steroidal anti-inflammatory drugs or aminoglycoside antibiotics may increase the risk of kidney injury. This may occur even at recommended drug doses and can be aggravated by dehydration or comorbidities. The podocin was presence in 4 horses receiving gentamicin. This may indicate that gentamicin may also damage podocytes to some extent. Renal toxicity of gentamicin is associated with its accumulation in proximal tubular cells, and correlates with drug concentration in the renal cortex [29]. The cytotoxic effect of gentamicin is complex and related to, among others, modulation of membrane enzyme activities and an increase in the production of reactive oxygen species, which leads to the loss of cell integrity and apoptosis [29]. Complications associated with the use of gentamicin have been observed mainly in young horses and foals, in dehydrated horses or in those simultaneously receiving other nephrotoxic drugs [28, 52]. In the present study, podocin was detected in the four youngest horses from the group of animals receiving gentamicin, which may confirm their exposure to nephrotoxic substances. It should also be taken into account that these animals received gentamicin due to various reasons. Therefore, co-existing factors could also be the cause of the obtained results. On the other hand, in the majority of the horses suspected of AKI, the authors did not detect podocin due to the use of gentamicin. Unfortunately, the gentamicin blood concentration was not assessed in any of the examined animals, therefore, it cannot be determined whether the presence of podocin was caused by an increased blood concentration of the drug. In the present study, the presence of podocin in the urine in the LC-MS-MRM examination was detected in five horses receiving NSAIDs and one of those horses had slightly elevated concentration of serum creatinine and urinary GGT. Renal toxicity associated with the use of phenylbutazone has been reported in horses and causes renal crest necrosis [25, 30, 31, 53]. The nephrotoxic effect of NSAIDs is elicited by a reduced renal plasma flow caused by a decrease in prostaglandins, which regulate vasodilation at the glomerular level, as well as the development of acute interstitial nephritis, which is characterized by the presence of an inflammatory cell infiltrate in the interstitium [54]. In patients with healthy kidneys, NSAID treatment may trigger a spectrum of nephritides, including tubular, interstitial, or tubulointerstitial nephritis, chronic interstitial nephritis with papillary necrosis and tubulointerstitial nephritis combined with the nephrotic syndrome [55]. The effect of NSAIDs on the renal function is most often compounded by other conditions or dehydration [25, 53]. In the group of horses at risk for AKI, the presence of podocin may be the result of both: the effect of a factor damaging the podocytes, as well as a physiological basis, due to the detection of podocin also in healthy horses. The lack of detection of podocin in some horses at risk of AKI, unfortunately, does not fully exclude the occurrence of subclinical AKI located outside the glomerulus.

The second method used in the study was a species-specific ELISA test to evaluate the podocin concentration. Immunoassays are considered the “gold standard” for protein quantification since they are based on highly specific interactions between the protein of interest and a targeted antibody. Moreover, some immunoassays allow the detection of very low amounts of proteins [16, 56, 57]. In the present study, urinary levels of podocin in healthy horses were lower than those reported in humans and dogs [5, 16, 23, 24]. As in the case of qualitative assessment, 4 healthy horses had a concentration of podocin above cut-off value, which may confirm the occurrence of physiological podocyturia in this species. Podocin has significantly higher values in horses with clinical kidney dysfunction compared to other examined horses, and the mean results were similar to those obtained in people with normo- and micro-albuminemia, but lower than in people with macro-albuminemia and evident kidney disease [23, 24]. No gradual increase in podocin was seen with disease progression in the analysis of the podocin values for horses at various stages of AKI. Despite the highest mean values of podocin obtained in the group of horses classified as VAKI 3, the VAKI stage 0 had higher values than stage 1 and 2. It should be noted that only some of the horses had a concentration of podocin above the cut-off value, while the remaining values did not indicate injury. Low levels of podocin in the AKI horses may be due to podocyte depletion during illness or a smaller participation of glomerular damage in the cause of kidney dysfunction. It has been proven that there is a mechanism for the progression of glomerular diseases, independent of the damage and reduction of podocyte numbers [58]. In the horses at risk for AKI, the podocin concentration was higher than in the healthy horses. However, the differences were statistically insignificant and still corresponded to the value assumed as normal in humans. Interestingly, the results obtained using the ELISA test showed no correlation with blood and urine results, except for a low positive correlation with the UPC and FENa value, which differs from the results cited earlier.

The presented studies showed substantial compatibility between LC-MS-MRM and ELISA. The LC-MS-MRM method was more likely to give positive results than ELISA and the ELISA test was more effective at excluding kidney dysfunction than confirming it. In the human LC-MS-MRM method, the limit of quantification was 0.39 ng/mL [16]. After analyzing the individual results obtained in the examined horses, this limit value was similar. Such a low detection threshold can lead to finding not only sick individuals, but also those with physiological podocyturia. Studies detecting other proteins and comparing both methods have shown that ELISA displayed less accuracy than LC-MS-MRM at lower concentrations of the tested substance, and its use in such cases can be problematic [59, 60]. Verbal reports and exchange of experience with human medicine doctors showed a similar problem in the case of human podocin and the use of ELISA and LC-MS-MRM methods. Recent evidence suggested that podocin may exist in a canonical, well-studied large isoform and an ill-defined short isoform [61]. In commercially available ELISA tests antibodies are not always specific to the targeted protein or may not discriminate protein isoforms and as a result, may under- or overestimate protein levels [62]. Another explanation can be found in selected peptide fragments used in the LC-MS-MRM analysis, which may correspond to another protein present in the body of the horse, which is not a podocin. The use of one of the tests in clinical practice to assess podocin in horses still has some limitations. The ELISA test is commercially available, simpler to perform, but an appropriate number of samples should be collected to ensure that the wells are not wasted. Assessment with LC-MS-MRM requires access to specialized equipment as well as experience in proper sample preparation, measurement, and interpretation.

The main limitation of the study was the impossibility to perform a biopsy in the examined animals. A histopathological examination of the kidney would allow to determine the type of changes occurring in the kidneys in horses with clinical kidney disease and horses at risk for AKI. It could confirm the true occurrence of glomerular injury in horses with podocyturia, as well as confirming the actual occurrence of physiological podocyturia in healthy animals. Also, a histopathological examination could confirm the absence of kidney disease in horses included in the group of healthy animals and at risk for kidney disease before the action of a potentially nephrotoxic factor. A biopsy was not performed because of lack of owner consent and consent of the ethics committee as such procedures carry the risk of complications. Also, due to the lack of generally available biomarkers enabling earlier exclusion of renal dysfunction in horses, the available gold standard, i.e., serum creatinine was the group inclusion criterion. As is well known, its increase only occurs with significant kidney damage, so some suspected and healthy horses may already have undiagnosed subclinical AKI. Classification of horses with renal dysfunction was based on a serum creatinine concentration without evaluation of the glomerular filtration rate, which could lead to the underestimation of the severity of the disease. Additionally, the concentration of some serum and urine parameters was not tested before the appearance of clinical signs, which could not entirely exclude some pre-existing loss of renal function that could have been exacerbated by a new acute disease process. An initial treatment of primary disease, sedation and general anesthesia may had some impact on blood and urine result. Therefore, further investigation involving more horses is needed for better understanding of podocyturia in horses and to improve diagnostic methods.

## Conclusion

Podocin may be a potential biomarker of glomerular injury and clinical kidney disease in horses, as well as in the assessment of subclinical cases with hypoperfusion-induced and drug-induced kidney injury. However, its usefulness may be limited due to the possibility of physiological podocyturia. LC-MS-MRM seems to be a more sensitive method for the detection of podocin than the ELISA test, and selected tryptic peptides of podocin appear detectable in horses. ELISA test is more effective at excluding the disease than confirming it. The use of one of the tests in clinical practice still has some limitations, and more research is needed to assess the clinical use of both methods because they reflect limited clinical, morphological and biochemical data. Also, the study showed the possibility of more frequent occurrence of glomerular injury in kidney pathologies in horses than it was previously thought and also the potential effect of NSAIDs, aminoglycosides, and hypoperfusion on glomerular damage.
